# Supplementary material for: An in vitro human vessel model to study Neisseria meningitidis colonization and vascular damages
Source: eLife. 2025 Dec 17;14:RP107813. doi: 10.7554/eLife.107813 (PMC12711198; doi:10.7554/eLife.107813)
Supplement: MDAR checklist [file elife-107813-mdarchecklist1.pdf]

Materials Design Analysis Reporting (MDAR)
Checklist for Authors

The MDAR framework establishes a minimum set of requirements in transparent reporting mainly applicable to studies in the life sciences.

eLife asks authors to **provide detailed information within their article** to facilitate the interpretation and replication of their work. Authors can also upload supporting materials to comply with relevant reporting guidelines for health-related research (see EQUATOR Network), life science research (see the BioSharing Information Resource), or animal research (see the ARRIVE Guidelines and the STRANGE Framework; for details, see *eLife*'s Journal Policies). Where applicable, authors should refer to any relevant reporting standards materials in this form.

For all that apply, please note **where in the article** the information is provided. Please note that we also collect information about data availability and ethics in the submission form.

Materials:

**Newly created materials**	**Indicate where provided: section/figure legend**	**N/A**
The manuscript includes a dedicated "materials availability statement" providing transparent disclosure about availability of newly created materials including details on how materials can be accessed and describing any restrictions on access.		
Poly-dimethyl-siloxane (PDMS, Silgard 184; Dow Chemical)	Figures 1-6 and associated figures supp.; Results.	
FujiFilm rat tail collagen I (LabChem Wako, Collagen-Gel Culturing Kit)	Figure 1 and Figure 1-figure supp. 1; Results.	
High concentration rat tail collagen I (Corning, #354249)	Figures 1-6 and associated figures supp; Results.	
UV-laser (MOPA- 1134 355, 500 mW, 10 kHz)	Figure 1 and Figure 1-figure supp. 1; Results.	

**Antibodies**	**Indicate where provided: section/figure legend**	**N/A**
For commercial reagents, provide supplier name, catalogue number and RRID, if available.		
Maleimide Alexa FluorTM 488 (ThermoFisher, #A10254)	Figure 1-figure supp. 1	
E-selectin (CD62E clone P2H3, Thermofisher Scientific, #12-0627-42)	Figure 6 and Figure 6-figure supp. 1; results	
Phalloidin (Alexa Fluor 488, Thermofisher, #A12379)	Figures 1, 3-6 and Figure 1-figure supp. 1 and Figure 2-figure supp. 1; Results	
Anti-human PECAM antibody (Ultra-LEAF™ Purified anti-human CD31 Antibody, Biolegend #303143 - conjugated with	Figure 2-figure supp.1; Results	

Dy-1311Light Antibody Labeling Kits, Termofisher #53044)		
Anti-human collagen IV antibody (Alexa Fluor 642, Clone 1042, eBioscience #51-9871-82)	Figure 2-figure supp.1; Results	
Rabbit anti-VE-cadherin (Abcam #ab33168) + Alexa Fluor 555-conjugated donkey anti-rabbit antibody (Ab-1316 cam #ab150074)	Figure 2; Results	
Hoechst (33362 trihydrochloride trihydrate, Invitrogen #H3570)	Figure 2 and Figure 2-figure supp.1; Results	
Dextran 70 or 150 kDa (FITC, Sigma)	Figure 2 and Figure 4; Results	
Actin (SPY555-actin, #CY-SC202)	Figure 6 and Figure 6-figure supp. 1; Results	
Dylight550-conjugated anti-mouse Ly-6G (LEAF purified anti-mouse Ly-6G, clone 1A8, Biolegend, #1276201407 with DyLight550 Antibody Labelling Kit, ThermoFisher Scientific, #84530)	Figure 6 and Figure 6-figure supp. 1; Results	

**DNA and RNA sequences**	**Indicate where provided: section/figure legend**	**N/A**
Short novel DNA or RNA including primers, probes: Sequences should be included or deposited in a public repository.<br><br>plasmid pmScarlet-I-C1 (Addgene #85044)<br>plasmid PacI_mScar_F<br>plasmid XhoI_mScar_R	 <br><br>Mat. and Met.<br>Mat. and Met.<br>Mat. and Met.	

**Cell materials**	**Indicate where provided: section/figure legend**	**N/A**
Cell lines: Provide species information, strain. Provide accession number in repository OR supplier name, catalog number, clone number, OR RRID.<br><br>*N. meningitidis* strains derived from the 8013 serogroup C strain (http://www.genoscope.cns. 1041 fr/agc/nemesys)	 <br><br><br>Figures 1-6 and associated figures supp.; Results.	
Primary cultures: Provide species, strain, sex of origin, genetic modification status.<br><br>Primary human umbilical endothelial cells (HUVECs) Lonza (pooled donors, #C2519A)<br><br>Human peripheral blood samples collected from healthy volunteers through the ICAReB-Clin (Clinical Investigation platform)	 <br><br>Figures 1-6 and associated figures supp.; Results.<br><br>Figure 6 and Figure 6-figure Supp. 1; Results	

Experimental animals	Indicate where provided: section/figure legend	N/A
Laboratory animals or Model organisms: Provide species, strain, sex, age, genetic modification status. Provide accession number in repository OR supplier name, catalog number, clone number, OR RRID.<br><br>SCID/Beige (CB17.Cg-PrkdcscidLystbg-J/Crl)	Figure 1-4 and 6; and associated figures supp.; Results.	
Animal observed in or captured from the field: Provide species, sex, and age where possible.<br><br>SCID/Beige (CB17.Cg-PrkdcscidLystbg-J/Crl)	Figure 1-4 and 6; and associated figures supp.; Results.	

Plants and microbes	Indicate where provided: section/figure legend	N/A
Plants: provide species and strain, ecotype and cultivar where relevant, unique accession number if available, and source (including location for collected wild specimens).		X
Microbes: provide species and strain, unique accession number if available, and source.		X

Human research participants	Indicate where provided: section/figure legend) or state if these demographics were not collected	N/A
If collected and within the bounds of privacy constraints report on age, sex, gender and ethnicity for all study participants.		**X**

Design:

Study protocol	Indicate where provided: section/figure legend	N/A
If the study protocol has been pre-registered, provide DOI. For clinical trials, provide the trial registration number OR cite DOI.<br><br>Philippe Esterre, *et al.* The icareb platform: A human biobank for the institut Pasteur and beyond. Open Journal of ***Bioresources***, 2020. doi: 10.5334/ojb.66	Figures 1-6 and associated figures supp.; Results.	

Laboratory protocol	Indicate where provided: section/figure legend	N/A
Provide DOI OR other citation details if detailed step-by-step protocols are available.<br><br>Christophe Rusniok, *et al.* Nemesys: a biological resource for narrowing the gap between sequence and function in the human pathogen neisseria meningitidis. ***Genome Biology***, 10(10):R110, Oct 2009. ISSN 1474-760X. doi: 10.1186/gb-2009-10-10-r110.<br><br>Arthur Charles-Orszag, *et al.* Adhesion to nanofibers drives cell membrane remodeling through one-dimensional wetting. ***Nature Communications***, 9(1):4450, Oct 2018. ISSN 2041-1723. doi: 10.1038/s41467-018-06948-x.<br><br>Daria Bonazzi, *et al.* Intermittent pili-mediated forces fluidize neisseria meningitidis aggregates promoting vascular colonization. ***Cell***, 174(1):143–155.e16, 2018. ISSN 730 0092-8674. doi: https://doi.org/10.1016/j.cell.2018.04.010	Figures 1-6 and associated figures supp.; Mat. and Met.<br><br>Figures 1-6 and associated figures supp.; Mat. and Met.<br><br>Figures 1-6 and associated figures supp.; Mat. and Met.	

Experimental study design (statistics details) *		
**For in vivo studies: State whether and how the following have been done**	**Indicate where provided: section/figure legend. If it could have been done, but was not, write "not done"**	**N/A**
Sample size determination		
Randomisation		
Blinding		
Inclusion/exclusion criteria		

Sample definition and in-laboratory replication	Indicate where provided: section/figure legend	N/A
State number of times the experiment was replicated in the laboratory.<br><br>All experiments have been replicated at least three times.	Figure 1-6; and associated figures supp.	
Define whether data describe technical or biological replicates.<br><br>Described data are technical and biological replicates.	Figure 1-6; and associated figures supp.	

Ethics	Indicate where provided: section/submission form	N/A
Studies involving human participants: State details of authority granting ethics approval (IRB or equivalent committee(s), provide reference number for approval.<br><br>Human-derived xenograft: All procedures were approved by the local ethical committee Comité d'Evaluation Ethique de l'INSERM IRB 00003888 1250 FWA 00005881, Paris, France Opinion: 11048.<br><br>Healthy donor – blood collection: CoSImmGEn cohort (Clinical trials NCT 03925272), after approval of the 1389 CPP Ile-de-France I Ethics Committee (2011, Jan 18th)	Mat. And Met.<br><br>Mat. And Met.	
Studies involving experimental animals: State details of authority granting ethics approval (IRB or equivalent committee(s), provide reference number for approval.<br><br>Mice animal experimentations: All experiments were performed in agreement with guide lines established by the French and European regulations for the care and use of laboratory animals and approved by the Institut Pasteur Committee on Animal Welfare (CETEA) under the protocol code CETEA 2018-0022	Mat. And Met.	
Studies involving specimen and field samples: State if relevant permits obtained, provide details of authority approving study; if none were required, explain why.		X

Dual Use Research of Concern (DURC)	Indicate where provided: section/submission form	N/A
If study is subject to dual use research of concern regulations, state the authority granting approval and reference number for the regulatory approval.		

Analysis:

Attrition	Indicate where provided: section/figure legend	N/A
Describe whether exclusion criteria were pre-established. Report if sample or data points were omitted from analysis. If yes, report if this was due to attrition or intentional exclusion and provide justification.		

No exclusion of data point has been performed. We kept all outliers in analysis data.	Figure 1-6 and associated figures supp.; Results; Mat. and Met.	

**Statistics**	**Indicate where provided: section/figure legend**	**N/A**
Describe statistical tests used and justify choice of tests.<br><br>All statistical tests are based on Wilcoxon method, a non-paired for non-gaussian population test (<1000 replicates).<br><br>In each figure, the individual data are displayed and the mean ± SD are always defined.<br><br>The p-values are indicated in each figure. The corresponding star-based legend is displayed as : p-values > 0.05: n.s., 0.05 < p-values < 0.01: *, 0.01 < p-values < 0.005: **, 0.005 < p-values < 0.001: ***, p-values < 0.001: ****.	Figure 1-6 and associated figures supp.; Mat. and Met.<br><br>Figure 1-6 and associated figures supp.<br><br>Figure 1-6 and associated figures supp.; Results; Mat. and Met.	

**Data availability**	**Indicate where provided: section/submission form**	**N/A**
For newly created and reused datasets, the manuscript includes a data availability statement that provides details for access (or notes restrictions on access).		X
When newly created datasets are publicly available, provide accession number in repository OR DOI and licensing details where available.	Gitlab.pasteur.fr/wong/photoablationvessel2025	
If reused data is publicly available provide accession number in repository OR DOI, OR URL, OR citation.		X

**Code availability**	**Indicate where provided: section/figure legend**	**N/A**
For any computer code/software/mathematical algorithms essential for replicating the main findings of the study, whether newly generated or re-used, the manuscript includes a data availability statement that provides details for access or notes restrictions.		X

Where newly generated code is publicly available, provide accession number in repository, OR DOI OR URL and licensing details where available. State any restrictions on code availability or accessibility.		X
If reused code is publicly available provide accession number in repository OR DOI OR URL, OR citation.		X

Reporting:

The MDAR framework recommends adoption of discipline-specific guidelines, established and endorsed through community initiatives.

**Adherence to community standards**	**Indicate where provided: section/figure legend**	**N/A**
State if relevant guidelines (e.g., ICMJE, MIBBI, ARRIVE, STRANGE) have been followed, and whether a checklist (e.g., CONSORT, PRISMA, ARRIVE) is provided with the manuscript.		X

---

* We provide the following guidance regarding transparent reporting and statistics; we also refer authors to Ten common statistical mistakes to watch out for when writing or reviewing a manuscript.

Sample-size estimation

- You should state whether an appropriate sample size was computed when the study was being designed
- You should state the statistical method of sample size computation and any required assumptions
- If no explicit power analysis was used, you should describe how you decided what sample (replicate) size (number) to use

Replicates

- You should report how often each experiment was performed
- You should include a definition of biological versus technical replication
- The data obtained should be provided and sufficient information should be provided to indicate the number of independent biological and/or technical replicates
- If you encountered any outliers, you should describe how these were handled
- Criteria for exclusion/inclusion of data should be clearly stated
- High-throughput sequence data should be uploaded before submission, with a private link for reviewers provided (these are available from both GEO and ArrayExpress)

Statistical reporting

- Statistical analysis methods should be described and justified
- Raw data should be presented in figures whenever informative to do so (typically when N per group is less than 10)
- For each experiment, you should identify the statistical tests used, exact values of N, definitions of center, methods of multiple test correction, and dispersion and precision measures (e.g., mean, median, SD, SEM, confidence intervals; and, for the major substantive results, a measure of effect size (e.g., Pearson's r, Cohen's d)
- Report exact p-values wherever possible alongside the summary statistics and 95% confidence

intervals. These should be reported for all key questions and not only when the p-value is less than 0.05.

Group allocation

- Indicate how samples were allocated into experimental groups (in the case of clinical studies, please specify allocation to treatment method); if randomization was used, please also state if restricted randomization was applied
- Indicate if masking was used during group allocation, data collection and/or data analysis
